# Incomplete intestinal obstruction as the possible main complaint in Behcet’s disease after surgery for recurrent abdominal aortic pseudoaneurysms: a case report and literature review

**DOI:** 10.1186/s12872-018-0977-z

**Published:** 2018-12-17

**Authors:** Fang Jiang, Hui Xiang, Zhi-Yong Peng

**Affiliations:** Department of Critical Care Medicine, Zhongnan Hospital of Wuhan University, 169 Dong′hu Road, Wuhan, 430071 Hubei People’s Republic of China

**Keywords:** Behcet’s disease, Recurrent abdominal aortic pseudoaneurysm, Incomplete intestinal obstruction

## Abstract

**Background:**

Behcet’s disease (BD) is a systemic vasculitis characterized by oral and genital aphthosis, and ocular and skin lesions. The disease is involved in vascular, gastrointestinal, and central nervous systems. Vasculitis may exacerbate fatal problems, such as anastomotic pseudoaneurysms. If the mesenteric vessels are involved, severe abdominal symptoms such as intestinal obstruction may occur.

**Case presentation:**

This case report describes a young female patient who suffered from BD with recurrent abdominal aortic pseudoaneurysms, as well as deep venous thrombosis and subsequent complications of incomplete intestinal obstruction. This patient first underwent stent grafting, which was followed by rupture of two newly formed anastomotic pseudoaneurysms within six months. Emergency open surgical repair (OSR) was then performed on the ruptured pseudoaneurysms. Thrombosis and incomplete ileus occurred five months after surgery. This case was unique due to the presence of incomplete intestinal obstruction being the possible main complaint for a patient with Behcet’s disease, and it is the first ever case to be reported.

**Conclusion:**

Intestinal obstruction may present as the possible main complaint in BD. Careful and attentive strategy should be carried out to prevent fatal outcomes.

## Background

Behcet’s disease (BD) is a disease that involves complex multisystem disorders. However, the exact physiopathologic mechanism of BD remains unclear. Evidence shows that arterial and venous systems are involved in BD. Involvement of the venous systems results in the occurrence of thrombosis and superficial phlebitis. Involvement of the arterial systems result in aneurysm or pseudoaneurysm, stenosis, and occlusion [1]. The pseudoaneurysm, which evolves quickly, is prone to rupture and can become life-threatening. Therefore, it is crucial to diagnose and manipulate this fatal condition early. If the mesenteric vessels are involved, severe abdominal symptoms such as intestinal obstruction may occur [2]. However, such symptoms, especially those presenting as the main complaint, are easily misdiagnosed. Here, we report a rare case of a patient who presented with incomplete intestinal obstruction as the possible main complaint after a second surgery. We believe that an examination of this case will help the field make progress toward developing a common procedural strategy for treating such rare conditions.

## Case presentation

A 28-year-old female Chinese patient presented with the onset of acute continuous right abdomen pain, nausea and vomiting at the emergency department. On admission, abdominal dynamic computed tomography (CT) with a multislice detector row CT scanner showed several air-fluid levels in the enteric cavity, and the diagnosis was considered to be ileus.

The patient was diagnosed with BD four years ago. She had received medications regularly, including immunosuppressive therapy with oral prednisone (60 mg/day) and cyclophosphamide (100 mg/day).

**Fig. 1:**
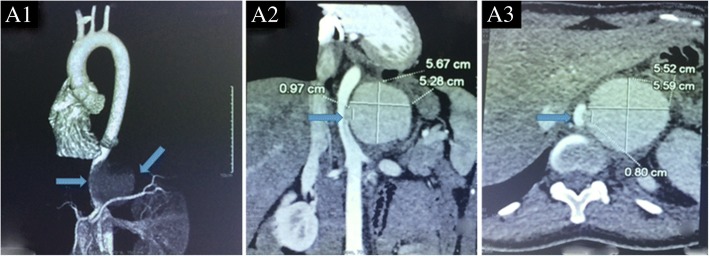
The aneurysm prior to intervention. A1:The aneurysm was located in the left junction of the thoracic and abdominal aorta. A2: The size of aneurysm was 5.67 cm*5.28 cm*0.97 cm. A3: The segmental block of the starting part of the celiac trunk was approximately 0.8 cm. Arrow pointed to the aneurysm

**Fig. 2:**
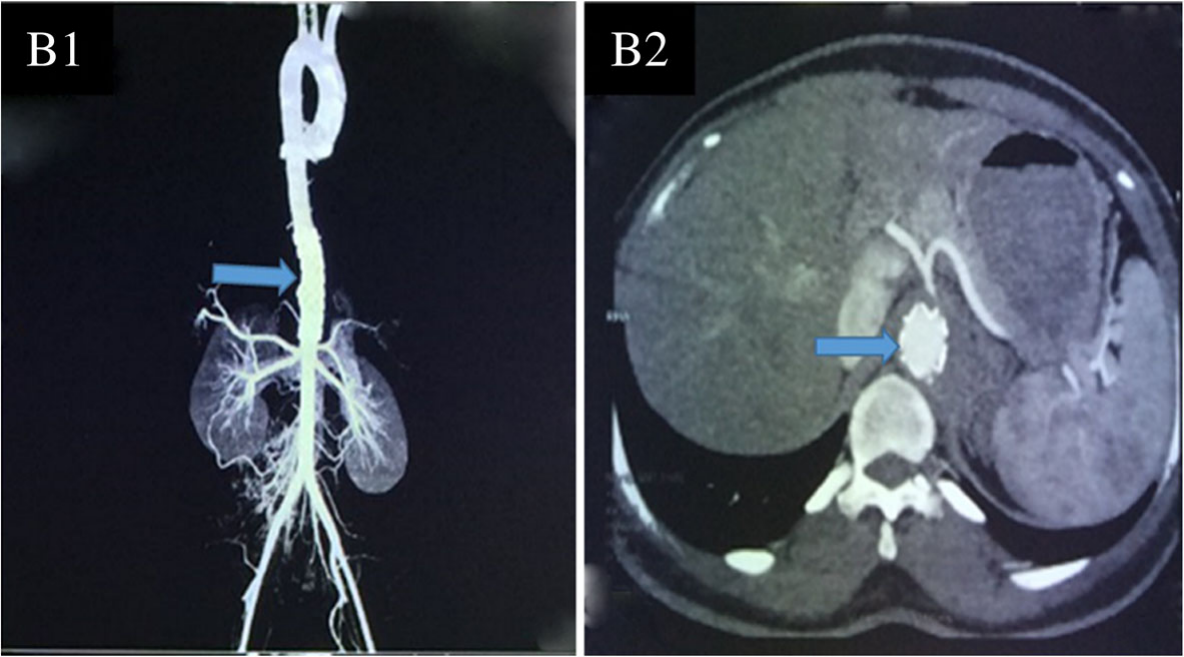
The aneurysm after intervention (B1, B2). The upper end was located in the cardia level, and the lower end in the upper edge of the superior mesenteric artery opening. Arrow pointed to the stent implantation site

**Fig. 3:**
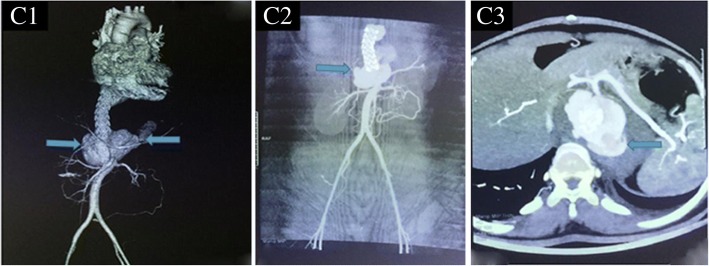
The hematoncus occured at 6 months after intervention. The size was 6.0*4.8 cm. The extravasation of contrast agent located in the opening of the renal artery, and the thickness of this mixed soft tissue mass was 3.7 cm

**Fig. 4:**
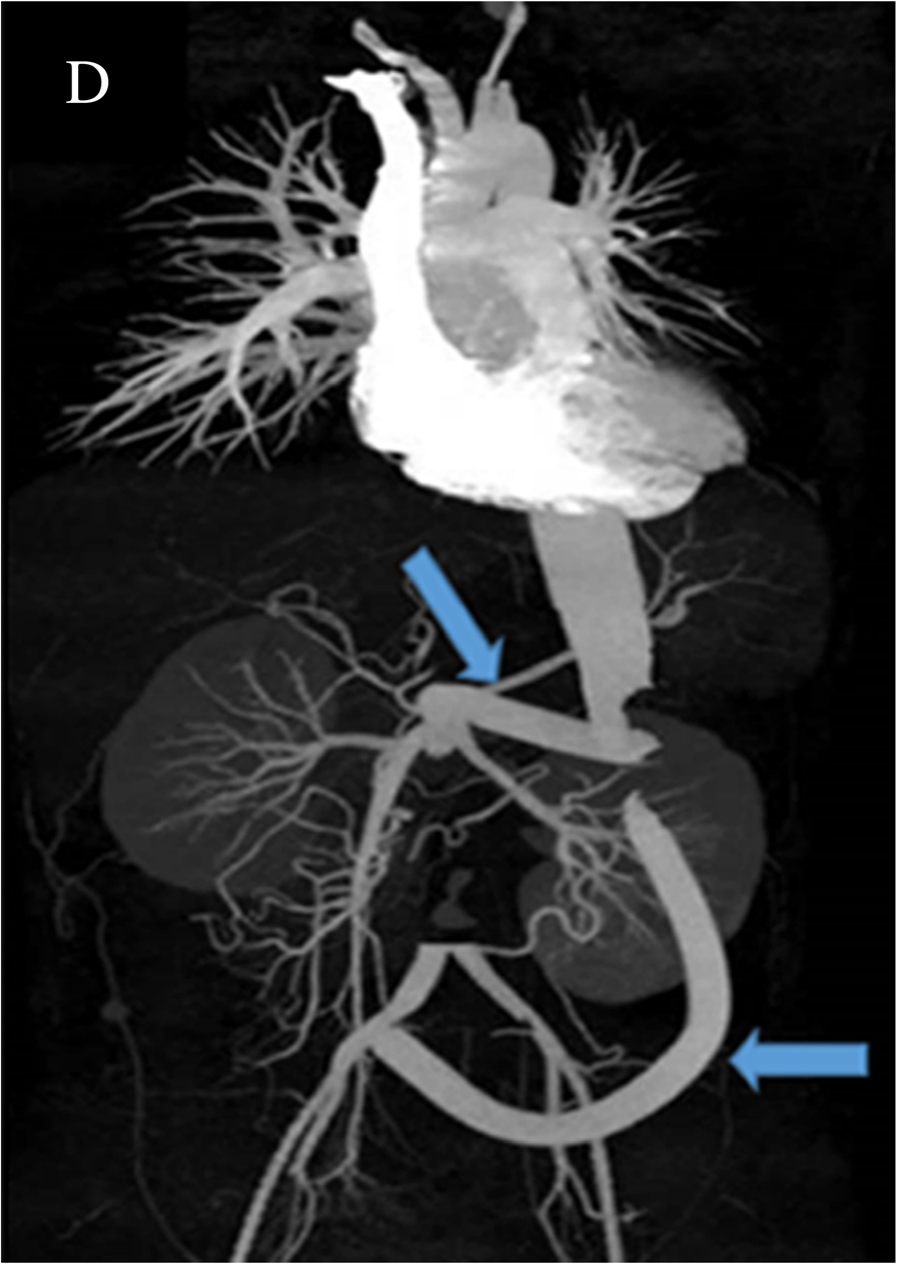
Implantation of artificial vascular in the thoracic and abdominal aorta. Two transplanted vessels were seen in picture D, one of which was connected to abdominal aorta, the other connected to right common iliac artery

The patient first presented with abdominal pain at the hospital. A computed tomographic angiography (CTA) was performed, which (Fig. 1) indicated an aneurysm of 5.67 cm*5.28 cm*0.97 cm located in the left junction of the thoracic aorta and abdominal aorta. An approximately 0.8 cm segment was found to block the starting part of the celiac trunk. Subsequently, a graft stent was implanted. Follow-up CTA (Fig. 2) showed no residual aneurysm. However, six months after the intervention, a rapidly growing mass was found in the lower abdomen, and the patient presented with nausea, vomiting, progressive and intermittent pain in the abdomen which radiated to her back. Before being admitted to the intensive care unit (ICU), the patient underwent CTA, which showed that there was a haematoma of approximately 6.0 × 4.8 cm in the abdomen. The extravasation of contrast agent was located in the opening of the renal artery, with a mixed soft tissue mass of 3.7 cm (Fig. 3) in the haemoperitoneum. The haemoglobin concentration decreased to 4.36 g/dL with abrupt hypotension (60/43 mmHg). The critical condition of the patient prompted the cardiac surgeons to perform open surgical repair (OSR) rather than a more conservative treatment. The patient was transferred to the ICU after the operation. Three days later, after her vital signs were stable, she was transferred back to the general ward. No recurrence of pseudoaneurysm was found during a follow-up of 15 days (Fig. 4). She continued receiving immunosuppressive therapy as usual. This course of treatment was decided based on previous studies showing that use of immunosuppressive therapy with cyclophosphamide and corticosteroids before and after surgical intervention could help prevent BD activation [3, 4].

**Fig. 5:**
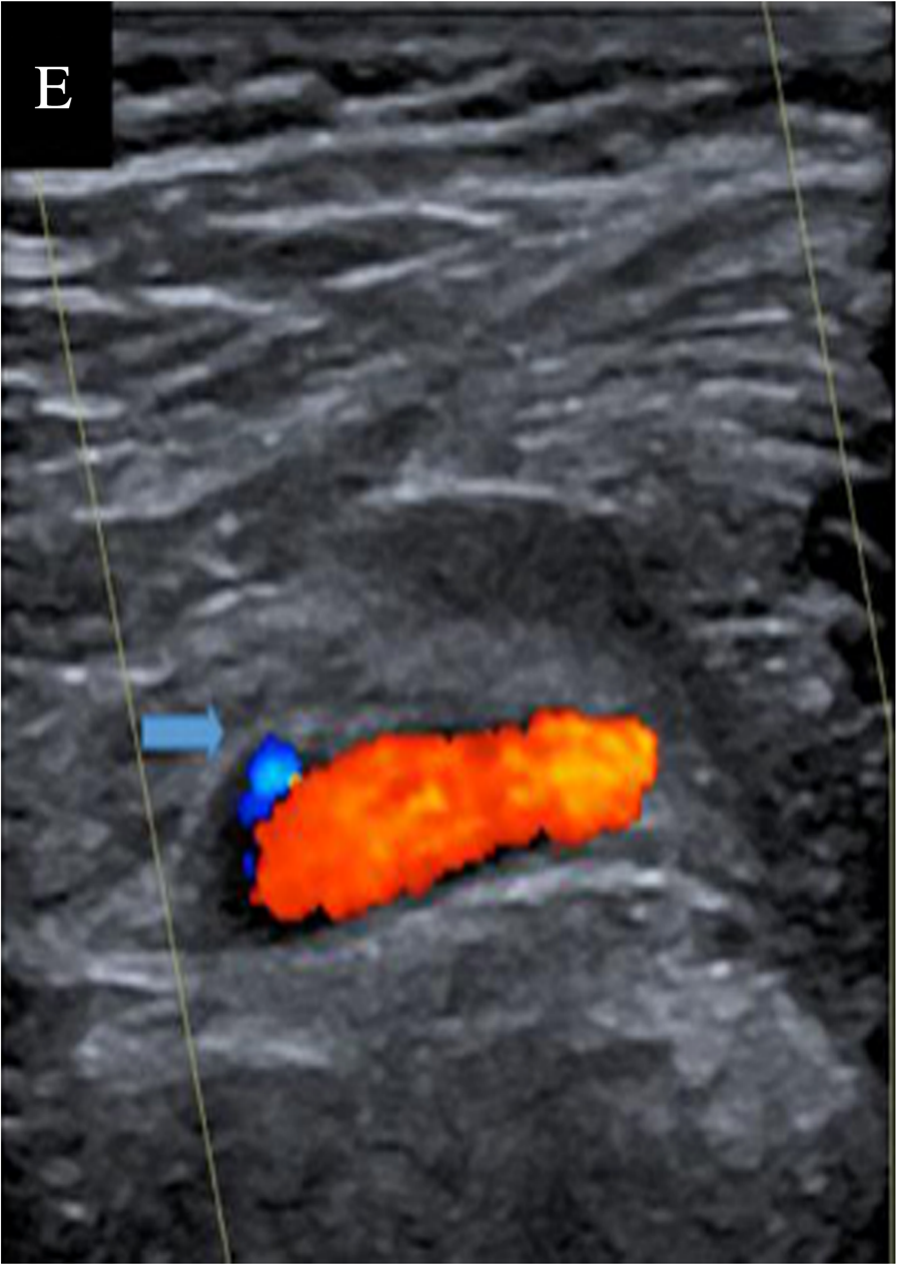
The deep venous thrombosis in the left popliteal vein. It was detected by doppler-ultrasound. Arrow pointed to the thrombosis

Ten months later, after the implantation of artificial vascularization in the thoracic and abdominal aorta, doppler-ultrasound indicated deep venous thrombosis in the left popliteal vein (Fig. 5). Therefore, the patient was treated with anticoagulant therapy using hypodermic injections of low-molecular-weight heparin at a daily dose of 4100 U.

**Fig. 6:**
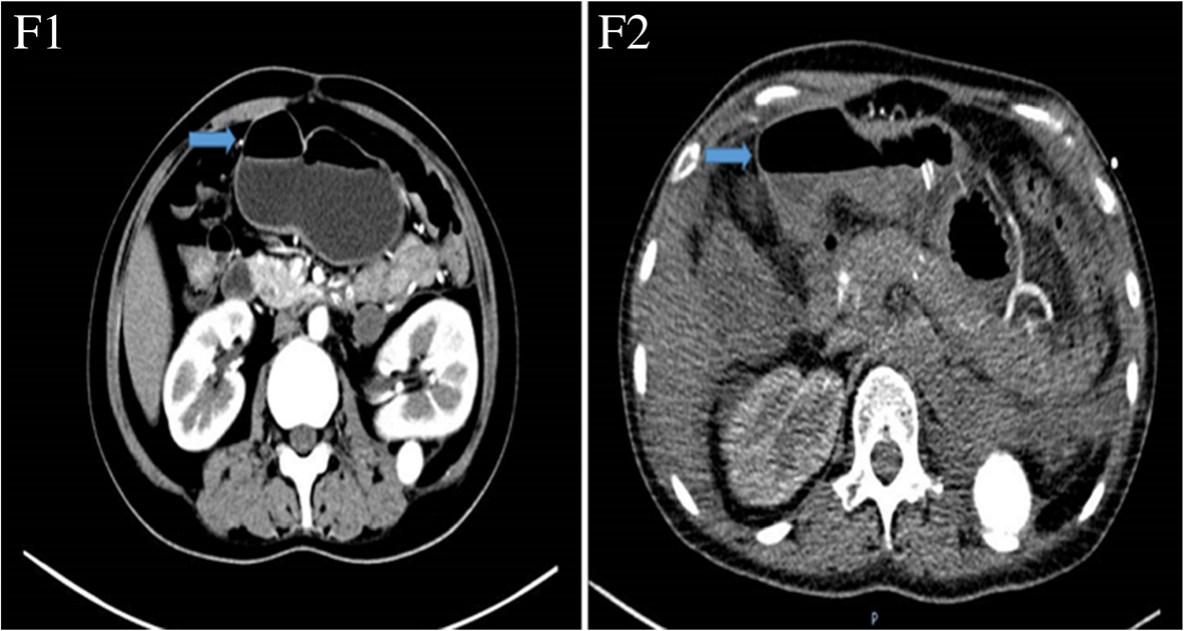
Air-fluid level (arrows). Detected by abdominal computed tomography

**Fig. 7:**
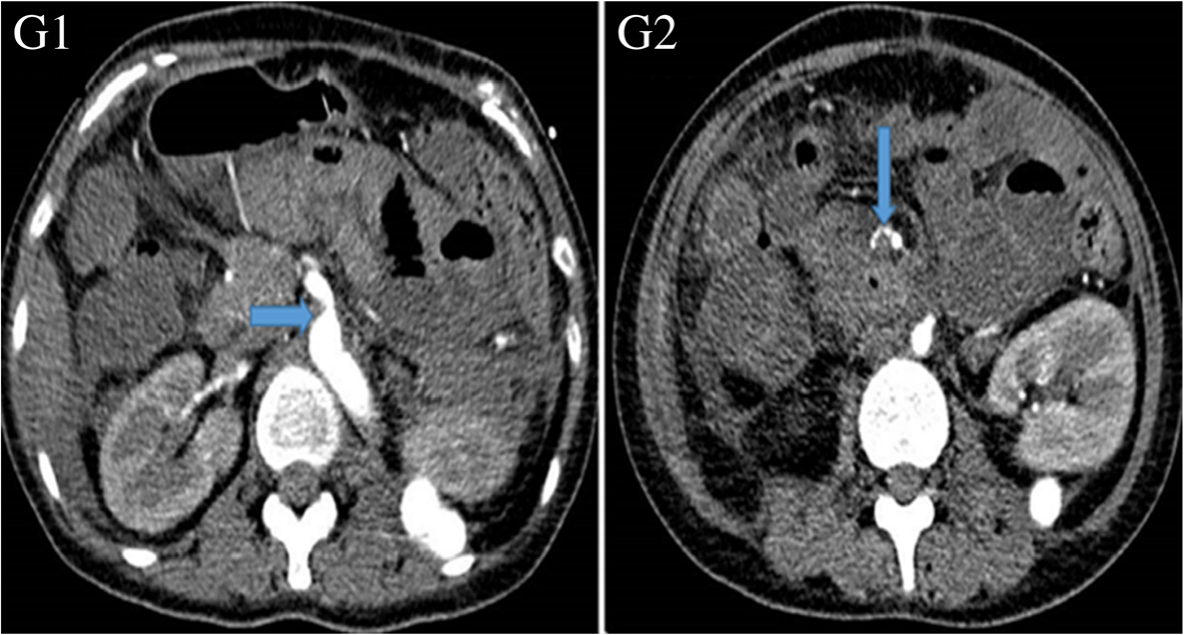
The root stenosis of celiac trunk and superior mesenteric artery. Detected by mesenteric artery angiography

One month later, the patient suffered from persistent right abdominal pain with nausea and vomiting accompanied by oral aphtha and genital ulcer. Abdominal CT showed the occurrence of an air-fluid level in part of the ileum and colon (Fig. 6). The diagnosis was determined to be an incomplete intestinal obstruction, which may have been caused by the previous aneurysms. Mesenteric artery angiography showed that the root of the celiac trunk and superior mesenteric artery were stenosed (Fig. 7). Then, conservative treatment was administered, such as fasting, gastrointestinal decompression and enema. Approximately 20 days later, the patient recovered well and was discharged from the hospital.

## Discussion and conclusions

Here we demonstrated a case of a patient with a diagnosis of BD who presented with incomplete intestinal obstruction as the possible main complaint. To the best of our knowledge, this is the first report of the presence of incomplete intestinal obstruction after a second surgery for BD.

Recent studies have reported that HLA-B51 is associated with a more severe disease and is related to familial BD [5]. Other studies have shown that polymorphism in the IL-21, IL-10, and IL-8 genes and in the tumour necrosis factor-(TNF-)alpha-1031C allele are correlated with the pathogenesis of BD [6–11]. The role of the IL-23/IL-17 axis has been found to be associated with the onset of BD [12, 13]. Meanwhile, delayed-type hypersensitivity to infections such as streptococcus species (especially sanguis) has also been proposed to be an important factor in inducing BD [14]. Hematological diseases such as coagulation abnormalities and platelet overactivity may play additional roles in the development of BD [15–17]. Nitric oxide (NO) accumulation after induction by interferon-gamma is also related to the activation of BD [18].

BD can be divided into two categories according to the pathological mechanism: occlusive and lesion. Although venous involvement is the most common vasculo-BD complication, Ketari et al. has been reported that arterial involvement is more common than venous involvement [19]. The most severe complication is aneurysm formation and rupture. Approximately 60% of reported arterial lesions associated with BD are aneurysms [20]. The aorta is the most commonly affected artery followed by the pulmonary artery. Aneurysms occur more frequently in the abdominal aorta than in the thoracic aorta [21–23].

Surgical repair is the standard treatment for aneurysms with dilatation, stenosis, or obstruction, while alternative treatments involve minimally invasive endovascular therapy for aneurysm repair [22, 24, 25]. Shen et al. reported that both open surgery and endovascular repair were safe and effective for treating aortic pseudoaneurysm in BD [26]. Furthermore, Naganuma et al. reported that ulcer lesions in the ileum and colon, which could lead to intestinal obstruction, were found in surgical patients with intestinal BD [27].

In our report, it was challenging to diagnose the patient who presented with initial symptoms of abdominal pain and vomiting. The differential diagnoses included pseudoaneurysm, ileus, new recurrent arterial aneurysm, mesenteric arterial embolism and so on. To obtain an accurate diagnosis, abdominal computed tomography and angiography were performed. CT/CTA results showed that the patient suffered from intestinal obstruction. The cause may be related to chronic vascular lesions, which could finally lead to an insufficient blood supply in the mesenteric artery. Literature review demonstrated that intestinal obstruction presented with BD in three cases. Of them, two cases of BD that presented with abdominal aortic aneurysm were diagnosed with ileus after stent graft interposition [28]. One patient with BD suffered from complete obstruction 6 years after several operations. The operations included an aortobifemoral bypass grafting for an abdominal aortic aneurysm [2] (Table 1). However, incomplete intestine obstruction was not reported as the main complaint for these patients. Our case report is the first to present incomplete intestinal obstruction as the possible main complaint of BD. Unfortunately, there are no certain guidelines for how to prevent and treat intestinal obstruction caused by BD. Thus, prompt differential diagnosis and management are essential for future treatment.

**Table 1:** Characteristics of lesion, treatment and outcome for patients complicated with intestinal obstruction from literature review

Patient	Location	symptom	Treatment (graft size: mm & type)	Complication	Immuno-suppresant	Recurrence	Follow up	Result
38 yrs. male	Infrarenal	C. Rupture	Interposition (Polytetrafluoroethylene) 16 × 8, bifurcated	Incomplete Intestinal Obstruction	Prednisolone + Colchicines	None	98 M	Alive
31 yrs. male	Suprarenal	Rupture	Patch (Polytetrafluoroethylene)	Incomplete Intestinal Obstruction	Colchicines + Azathioprine	18 M	43 M	Alive
50 yrs. male	Suprarenal	vascular thrombosis	Stent graft	Complete Intestinal Obstruction	Not clear	72 M	5 M	Alive

Considering the paucity of data available, the management of patients with intestinal obstruction should be individualized, and a multidisciplinary approach should be taken. Differential diagnosis and management are essential when addressing this fatal problem.

Technologies such as abdominal computed tomography and angiography are recommended resources when determining differential diagnosis. Finally, if intestinal obstruction is confirmed with the diagnosis, appropriate measures (surgical or nonsurgical treatment) to treat this disease should be taken.

## Abbreviations

BD: Behcet’s disease
CT: Computed tomography
CTA: Computed tomographic angiogram
ICU: Intensive care unit
NO: Nitric oxide
OSR: Open surgical repair
